# Pathophysiology of Penetrating Captive Bolt Stunning in Horned and Polled Sheep and Factors Determining Incomplete Concussion

**DOI:** 10.3390/vetsci12010053

**Published:** 2025-01-13

**Authors:** Troy John Gibson, Anne Lisa Ridler, Georgina Limon, Christopher Lamb, Alun Williams, Neville George Gregory

**Affiliations:** 1 Animal Welfare Science and Ethics Group, Department of Pathobiology and Population Sciences, Royal Veterinary College, Hawkshead Lane, Hatfield AL9 7TA, UK; 2 School of Veterinary Science, Massey University, 4410 Palmerston North, New Zealand; a.l.ridler@massey.ac.nz; 3 The Pirbright Institute, Woking GU24 0NF, UK; 4 Veterinary Epidemiology, Economics and Public Health Group, Department of Pathobiology and Population Sciences, Royal Veterinary College, Hawkshead Lane, Hatfield AL9 7TA, UK; 5 Independent Researchers, UK; clamb@btinternet.com (C.L.);; 6 Department of Veterinary Medicine, University of Cambridge, Cambridge CB2 3EN, UK; aw510@cam.ac.uk

**Keywords:** animal welfare, captive bolt, humane euthanasia, horned, magnetic resonance imaging, pathophysiology, polled, sheep

## Abstract

Penetrating captive bolt (PCB) is widely used for stunning and on-farm dispatch of livestock, yet its efficacy can vary, with the potential for animal welfare compromise. This study investigated the pathophysiology of PCB-induced trauma in horned and polled sheep (*Ovis aries*), focusing on factors contributing to incomplete concussion. Thirty-seven (*n* = 18 horned Scottish blackface and *n* = 19 polled North Country mule) mature ewes (aged 4–10 years) were shot with PCB with varying cartridge power and PCB modifications, followed by clinical assessment and post-mortem analysis using magnetic resonance imagining (MRI) and gross pathology. The results indicated that damage to the reticular activating system, bolt velocity and penetration depth are crucial for inducing irreversible unconsciousness, with depths less than 37 mm often resulting in incomplete concussion. MRI provided detailed insights into brain injuries, aligning well with gross pathological findings. This study highlights the importance of precise bolt placement and appropriate PCB configurations in ensuring humane outcomes, with MRI proving to be a valuable tool for assessing brain trauma in stunned animals. These findings enhance the understanding of effective stunning techniques and support improved welfare practices in livestock management.

## Simple Summary

The stunning of livestock prior to slaughter is intended to minimise animal suffering. This study investigated the use of penetrating captive bolt stunning in sheep, focusing on why this method sometimes fails to render animals unconscious and identifying the factors that influence its effectiveness. The behaviour of 37 horned and polled (unhorned) mature ewes (aged 4–10 years) was assessed after captive bolt stunning and associated with the extent of brain damage observed through brain imaging and physical examination. The findings revealed that damaging specific brain structures, the bolt’s speed, and its penetration depth are critical factors in inducing unconsciousness. Brain imaging provided detailed insights into brain trauma, complementing the findings from physical examinations. This study highlights the importance of accurate bolt placement during stunning. These findings enhance the understanding of captive bolt stunning mechanisms and could contribute to improving animal welfare during slaughter.

## 1. Introduction

Penetrating captive bolt (PCB) is a commonly used method for stunning animals for human consumption and on-farm dispatch of livestock species. It involves the firing of a retractable steel bolt into the head, which enters the cranial vault. Unconsciousness is induced by a combination of the delivered kinetic energy and trauma (focal and diffuse) to the brain and, in particular, the structures of the reticular activating system [1]. Yet, its efficacy can vary significantly, even within the same species. The pathophysiology of PCB injury, particularly when resulting in incomplete concussion, remains inadequately understood [2]. This lack of clarity poses significant welfare concerns, especially in horned and polled sheep (*Ovis aries*), where anatomical variations can influence the outcome of the procedure.

The use of PCB is not only critical in abattoirs but also during outbreaks of infectious animal diseases. During such outbreaks, it is sometimes necessary to cull large numbers of animals on farms over a short time period as part of control policies to prevent the spread of disease [3]. Methods requiring a two-stage process (e.g., stunning plus exsanguination or pithing) are not always practical in emergency situations. For instance, during the 2001 foot-and-mouth disease outbreak in the UK, there were anecdotal reports that secondary procedures (pithing or exsanguination) were not always performed after the initial shot, potentially leading to welfare issues. Gilliam et al. [4], Derscheid et al. [5], and Gibson et al. [3] investigated the use of PCB as a single-step killing method for adult cattle, mature calves and sheep, respectively, with all reporting that it can function as a single-step killing method in certain conditions. However, all three studies reported cases of incomplete concussion requiring the use of a secondary intervention (additional shots or chemical euthanasia). The humane dispatch of livestock in these scenarios is paramount to prevent further suffering and to control disease spread effectively. However, achieving a rapid and effective kill is complicated by PCB selection/maintenance, breed and intra-breed anatomical variation and the logistics of handling and restraining animals under field conditions.

Previous research has examined the efficacy of PCB and non-penetrating captive bolt (NPCB) stunning of sheep using time to undoubted unconsciousness/insensibility using spontaneous electroencephalogram (EEG) [6,7,8,9,10], evoked potentials [6,7,8], brainstem and cranial/spinal reflexes [3,10,11,12], and pathological assessment of traumatic brain injury (TBI) [3,13,14]. Together, this body of research highlights the challenges in ensuring complete irreversible unconsciousness in sheep using PCB and NPCB, particularly the importance of appropriate captive bolt gun/cartridge selection, bolt velocity and shot placement. However, there has been limited detailed research examining the underlying pathophysiology of captive bolt injury and the level of trauma required to produce irreversible concussion. Gibson et al. [3] examined this at the gross macroscopic level in polled and horned sheep, highlighting the importance of correct positioning and the influence of anatomy on the success of the captive bolt method. However, the variability in outcomes necessitates a deeper understanding of the factors contributing to incomplete concussion to improve animal welfare during stunning for human consumption or for disease control operations.

The primary aim of this study was to identify the pathophysiology of PCB trauma that results in incomplete concussion in horned and polled ewes, seeking to improve the efficacy of PCB. A secondary aim was to assess the extent to which gross macroscopic pathological examination of the fixed brain aligns with magnetic resonance imagining (MRI) to classify the degree of PCB TBI in different regions of the brain.

## 2. Materials and Methods

### 2.1. Animals and Experimental Design

Thirty-seven horned (Scottish blackface; *n* = 18) and polled (North Country mule; *n* = 19) ewes, aged between 4 and 10 years of age, were sourced from a commercial farm. Ewes were housed indoors and kept in accordance with normal husbandry practices. The study was conducted indoors in laboratory conditions, with only animals that were deemed in good physical condition with no physical signs of lameness included in the study. Prior to experimentation, the wool covering the ventral aspect of the neck was clipped to permit access to the jugular veins for catheterization or euthanasia purposes if required. The jugular vein was intravenously cannulated with a catheter (16 G, 3.25 inch, BD Angiocath, Becton Dickinson, UT, USA) and the cannula was secured in position with tape (Tensoplast, BSN Medical, South Africa). A bolus of approximately 170 u/kg sodium heparin (Multiparin, CP Pharmaceuticals Ltd., Wrexham, UK) and 49 mg/kg of contrast agent Gadoteric Acid (gadolinium) (Dotarem, Guerbet Laboratories, Solihull, UK) was administered intravenously via the jugular cannula 5 to 10 min prior to shooting.

Initially, all animals were shot with a 0.22 calibre Cash Special 2.5 grain (gr) PCB/cartridge combination (serial no. 2051, Accles & Shelvoke, Sutton Coldfield, UK) (Table 1). The ewes were shot into the cranial vault with a variety of positions and angles to simulate possible shooting styles (frontal, top of the head and poll); the muzzle was in contact with the head with all shots. Immediately after shooting, all animals were observed for clinical signs of consciousness/sensibility, including the presence or absence of immediate collapse, general spasm, leg flexion, righting reflex, rhythmic breathing, jaw muscle tension, heartbeat (palpation of the chest), corneal reflex, palpebral reflex, eyeball rotation, pupil dilatation, wrinkling of cornea, nystagmus, leg kicking and response to a hindleg cleat pinch [3,15,16,17,18]. Recordings were taken for 5 min after shooting or, if the heart was still beating, they were continued until the onset of cardiac arrest. Ewes were classified as conscious/sensible immediately after PCB shooting if either rhythmic breathing or failure to collapse was present and/or if two of more of the following parameters were present: positive corneal reflex, positive palpebral reflex, eyeball rotation and nystagmus. Shallow depth of concussion was defined as the presence of eyeball rotation, nystagmus or an intermittently positive corneal or palpebral reflex.

As there were no cases of failed shots or shallow depth of concussion in the first 25 sheep, the following alterations were made to the protocol to produce examples of reversible concussion: lower power cartridges (1 gr cartridges), alterations to the gun to reduce penetration depth (spacer: 18 mm spacer added to the outer aspect of the muzzle) and/or enlargement of the expansion chamber (1 mm travel: trimming of recuperator sleeve within the barrel to enlarge the expansion chamber by 1 mm). The rest of the ewes were shot using a combination of these modifications (Table 1). After shooting, any animals displaying signs of consciousness/sensibility and/or a shallow depth of concussion were immediately euthanized with an overdose of intravenous pentobarbitone sodium (Euthatal, Merial Animal Health Ltd., Harlow, UK).

Peak velocity of the PCB/modifications/cartridge combinations was tested with a custom-built velocity meter (Solutions for Research, Silsoe, Bedford, UK), as previously described by Gibson et al. [1]. Peak velocity was recorded and used to calculate the kinetic energy of the bolt using the equation *Kinetic energy* = (½ × *m*) × *v*^2^, where *m* = mass of bolt (kg), and *v* = peak velocity (m.s^−1^).

### 2.2. Magnetic Resonance Imaging (MRI)

Following the behavioural/reflex recording period and chemical euthanasia if required, individual animals were placed in the MRI scanner. The mean time to the first images was 545 ± (SD) 160 s after the shot. Sheep were placed in dorsal recumbency in the MRI, with a human spine coil in a 1.5 Tesla superconducting magnet (Intera, Philips Medical Systems, Best, The Netherlands). Care was taken to ensure that the head was positioned to prevent movement and displacement of brain and bone tissues in the captive bolt wound. Sagittal and transverse MRI images of the brain were acquired using T1-weighted and T2-weighted sequences. MRI images were 3.5 mm thick with 0.3 mm interspace. Transverse images were orientated perpendicular to the long axis of the bases cranii and medulla. Total imaging time varied from 40 to 50 min depending on the number of images acquired. Digitised scans were stored electronically for later analysis.

### 2.3. Gross Fixed Pathology and Histopathology

Following MRI imaging, both carotid arteries were cannulated with 18G IV Cannula (Abbocath-T, Hospira, Donegal Town, Ireland) for perfusion fixation of the brains in situ with buffered neutral formalin (10%) (Solmedia, Essex, UK). The canulae were connected to silicon tubing and intravenous drip sets, which were attached to a 5-litre reservoir, suspended 2 m above the carcass. Perfusion was gravity fed, with flow regulated via the intravenous drip sets, for 30 min. Following perfusion, the dorsal aspect of the cranium was removed with care taken not to damage the underlying brain. The entire head minus the jaw was placed in buffered neutral formalin for at least 30 days to minimise deformation of the brain and to ensure any intracranial haemorrhage was fixed in situ.

Fixed brains were then removed from their skulls and larger bone fragments removed from within the brain. The brains were then sliced, transversely, as 4 mm thick sections, starting at the cruciate sulcus and working rostrally and caudally to give 19–21 coronal slices of brain (depending on the length of brainstem present on the dissected-out brain, and any caudal displacements). For histopathological analyses, brain slices (either of whole cross-section or half cross-section) were dehydrated over 3–4 days and embedded in paraffin wax. Sections from the brainstem were cut at a thickness of 6 microns and stained with haematoxylin and eosin, cresyl violet, luxol fast blue-cresyl violet, Marsland and Glees silver stain and immunostained for amyloid precursor protein (APP). Brain slices and tissue sections were evaluated for location of captive bolt penetration, location and extent of haemorrhage, gross displacement of brain structures, and histological changes to neuronal cell bodies and axons.

### 2.4. Assessing Degree of Damage

Magnetic resonance and fixed gross pathology images were examined separately by the same two operators with the animal ID and treatment blinded. Severity of tissue damage to specific brain regions (occipital, temporal, parietal and frontal lobes, thalamus, midbrain, pons, medulla, cerebellum and spinal cord) was subjectively assessed as none (0%), mild (1–20%), moderate (21–49%) and severe (≥50%) [3,16,19]. In addition, skull thickness, soft tissue depth, point of shot entry relative to bregma (junction of the coronal and sagittal sutures on the top of skull), midline shift of brain structures and bolt penetration were measured (all in mm). Bolt penetration depth was determined from MRI imagines. Cranial factures were not assessed.

### 2.5. Statistical Analysis

Descriptive statistics were conducted and stratified by head type, PCB configuration and severity of tissue damage. The distribution of the data was examined and subsequently analysed with parametric (*t*-test) and non-parametric (Mann–Whitney U) tests where appropriate. PCB configurations were collapsed into a binary variable by joining all PCB with 1 gr cartridge into one variable (1 gr vs. 2.5 gr cartridge). Crude association between head type and ewes’ heads characteristics (i.e., soft tissue depth and skull thickness), head type and bolt penetration, and PCB configuration and bolt penetration was assessed using *t*-test. The relationships between skull thickness and bolt penetration, and soft tissue depth and bolt penetration, were assessed using linear regression.

Results from MRI and fixed gross pathology were tested for agreement using Cohen Kappa analysis. Weighted and unweighted Kappa estimates were calculated. The level of agreement was interpreted as: <0.20 poor, 0.21–0.40 fair, 0.41–0.60 moderate, 0.61–0.80 substantial, 0.81–1.00 very good.

For further statistical analysis, categorical variables representing severity of tissue damage were recategorized into binary variables. For this, moderate and severe damage were joined into one category (mild vs. moderate/severe). Similarly, ewes with signs of consciousness/sensibility and shallow depth of concussion were pooled and classed as incomplete concussion. The strength to which head type, PCB configuration, skull thickness, soft tissue depth, and bolt penetration were associated with incomplete concussion was determined using Fisher exact test or t-test as appropriate.

Data reduction techniques were utilised to describe the profile of tissue damage based on severity of damage classified by MRI and fixed gross pathology. As a first step, multiple corresponding analysis (MCA) was performed. MCA allows for a reduction in multivariate data by transforming correlated variables into a smaller number of synthetic uncorrelated factors, allowing for identification of patterns in complex datasets. Hierarchical cluster analysis (HCA) was then used to group animals into clusters according to their level of similarity in the factors created by MCA. Three separate analyses were considered: using gross pathology classification only; using MRI classification only; and using classification from both (gross pathology and MRI). Logistic regression was then used to assess the strength of association between incomplete concussion and the clusters identified by the MCA and HCA. Odds ratios were obtained as a measure of strength of association.

Statistical analysis was performed in R.3.6.1 (R Development Core Team 2017) using packages car, psych, FactoMineR, lme4 and odds ratio. For all analyses, *p* < 0.05 was used as the threshold for significance.

## 3. Results

### 3.1. Captive Bolt, Ewe Characteristics and Behavioural/Reflex Responses

**Figure 1:**
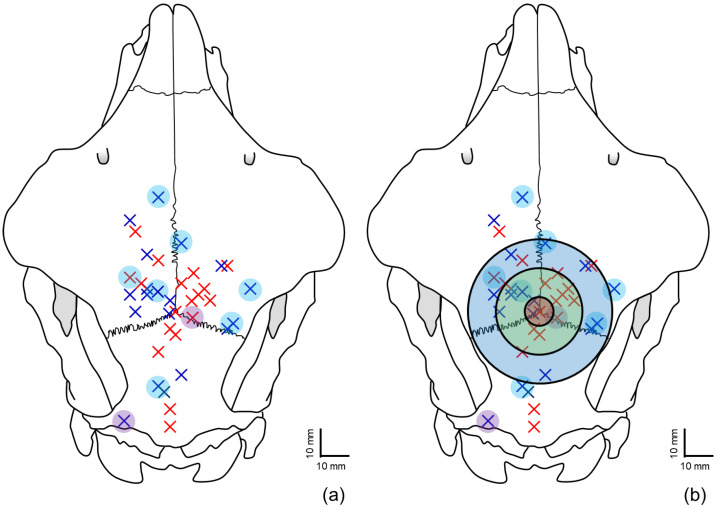
Penetrating captive bolt entrance positions for 37 ewes, depicted on one skull, relative to bregma. (**a**) Red crosses represent shots on horned ewes (*n* = 18), blue crosses represent shots on polled ewes (*n* = 19). Blue circles represent animals classed as conscious/sensible and purple circles represent animals showing shallow depth of concussion. Black rings in (**b**) delimit 5 mm, 15 mm, 25 mm and >25 mm relative to bregma.

Thirty-seven ewes were shot into the cranial vault in a variety of positions and angles to simulate different shooting styles (Figure 1). Around two-thirds of the shots were either 6 to 15 mm (*n* = 14; 37.8%) or 16 to 25 mm (*n* = 11; 29.7%) from bregma (Table 2).

Ewes were shot with a variety of PCB configurations and two cartridge types, as outlined in Table 1. Peak bolt velocity and kinetic energy for the PCB and 2.5 gr cartridge combination were 42.3 m.s^−1^ and 189 J, respectively. The PCB and 1 gr combination with or without the muzzle spacer had a peak bolt velocity of 27 m.s^−1^ and kinetic energy 78 J. Meanwhile, the addition of 1 mm of bolt travel and 1 gr cartridge plus spacer reduced peak bolt velocity to 21 m.s^−1^ and kinetic energy by 49 J.

Skull characteristics were similar, regardless of the head type, with no significant differences between soft tissue depth or skull thickness and head type (Table 2). A negative linear relationship was found between skull thickness and bolt penetration (r^2^ = 0.15, *p* = 0.02). There was a significant association between bolt penetration and PCB configuration used (*p* = 0.0003), but no difference was found between bolt penetration and head type (Table 2).

Almost all ewes (35; 94.6%) based on MRI had brain extrusion coming out of the top of the bolt hole, and half of the animals (18; 48.6%) had bone fragments displaced internally with a different path to the bolt. The overall proportion of animals displaying incomplete concussion was 24.3% (*n* = 9). Only one ewe that was shot with the 2.5 gr cartridge had rhythmic breathing. The 1 gr/spacer/travel configuration resulted in the highest proportion of cases of incomplete concussion (5/7; 71.4%) (Table 3 and Supplementary Materials S1 and S2). Eyeball rotation was observed in six animals post shooting but was not always associated with other signs of incomplete concussion.

### 3.2. MRI, Gross Fixed Pathology and Histopathology

Seven ewes (one horned and six polled) showed signs of consciousness/sensibility after the shot. Of these animals, four had no thalamus/brainstem (thalamus, midbrain, pons and medulla) damage, as assessed from MRI and fixed gross pathology. Meanwhile, of the remaining three animals, one had only mild damage in the thalamus (bolt penetration depth 18 mm) and the other had mild distortion in the midbrain and pons (bolt penetration depth 40 mm); this ewe was rhythmically breathing and had eyeball rotation after the PCB shot. The final ewe had moderate distortion of the thalamus and midbrain (bolt penetration depth 34 mm), with eyeball rotation and nystagmus but no other signs.

Two ewes (one horned, one polled) were classed as showing a shallow depth of concussion, having periods of eyeball rotation. The horned ewe had severe damage to the occipital lobe, mild to the temporal, midbrain/pons and none to the thalamus. For the polled ewe, the bolt had only superficially grazed the cerebrum and brainstem with damage, principally to the cerebellum (severe) pons (mild) and medulla (mild) from bone fragments acting as secondary missiles.

Of the ewes that were completely concussed with no signs of consciousness/sensibility, 94% (16/17) and 93% (13/14) of horned and polled ewes, respectively, had some level of macroscopic trauma to the thalamus, midbrain, pons and medulla. The remaining horned ewe only had trauma to the frontal (severe) and parietal (mild) lobes, while the remaining polled ewe had severe damage to the frontal lobe only. Severity of brain injury in each ewe (horned and polled ewes respectively) in relation to PCB configuration and signs of recovery is presented in the Supplementary Materials Tables S3 and S4. Based on MRI, in 95.6% of heads, there was a column of brain tissue extending out of the cavity.

Table 4 details the level of agreement between MRI and fixed gross pathology for specific regions of the brain. Overall MRI classified more regions with severe damage than gross pathology. The highest level of agreement was for direct damage to the paraflocculun (Kappa 0.61). There was moderate agreement for direct damage to the occipital, parietal and frontal lobes of the cerebrum, vermis, olfactory bulbs, hippocampus fimbria, corpus callosum splenium, midbrain, medulla and hippocampus fim, and fair agreement for direct damage to corpus callosum genu, lateral ventricles, thalamus, pineal, pons, corona radiata and internal capsule.

**Figure 2:**
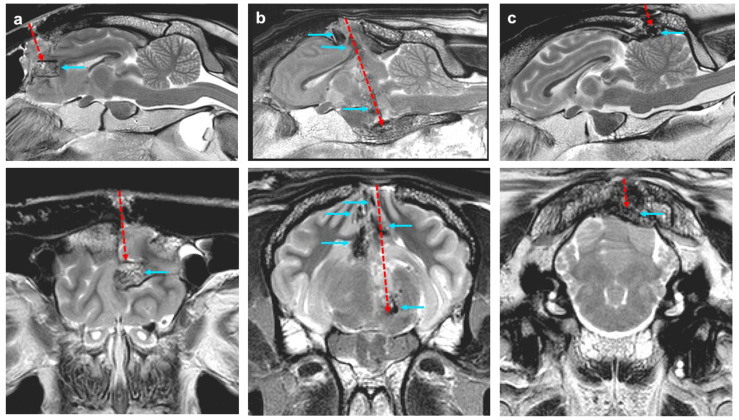
Sagittal and transverse MRI images of ewes shot with a PCB. (**a**) The ewe was incompletely concussed with the shot entering the rostral aspect of the frontal lobe with a large bone fragment lodged in the brain and tissue extending out towards the bolt entrance cavity. (**b**) Permanently concussed ewe with the shot just left of midline into the parietal/occipital lobes with the bolt going into the midbrain, with cerebellar coning. (**c**) Incompletely concussed ewe shot with a lower powered cartridge into the cerebellum, with a bone fragment depressing on the cerebellum. Red dashed arrows represent the trajectory and direction of the bolt, while blue arrows indicate the location of bone fragments within the brain.

Figure 2 displays example MRI images from three different ewes with different pathologies and clinical outcomes. Figure 2a shows sagittal and transverse MRI images of a ewe shot with the 1 gr cartridge, spacer and 1 mm of travel in the bolt. The bolt entered left of midline into the rostral aspect of the frontal lobe with a large bone fragment lodged in the frontal lobe and tissue extending out towards the bolt entrance cavity. The ewe in Figure 2b was shot just left of midline into the parietal/occipital lobes, with the bolt going into the midbrain. In this animal, there are multiple bone fragments in the region of the path of the bolt. Parietal/occipital lobe tissue is extending out towards the bolt entrance cavity in the skull, and there is serve cerebellum conning. Figure 2c shows a ewe that was shot with a 1 gr cartridge, spacer and 1 mm of bolt travel. This was shot in the occipital bone, with a bone fragment depressing onto the surface of the cerebellum, causing minimal physical damage. The ewes in Figure 2a,c showed signs of incomplete concussion, while the ewe in Figure 2b was permanently concussed, leading to death.

**Figure 3:**
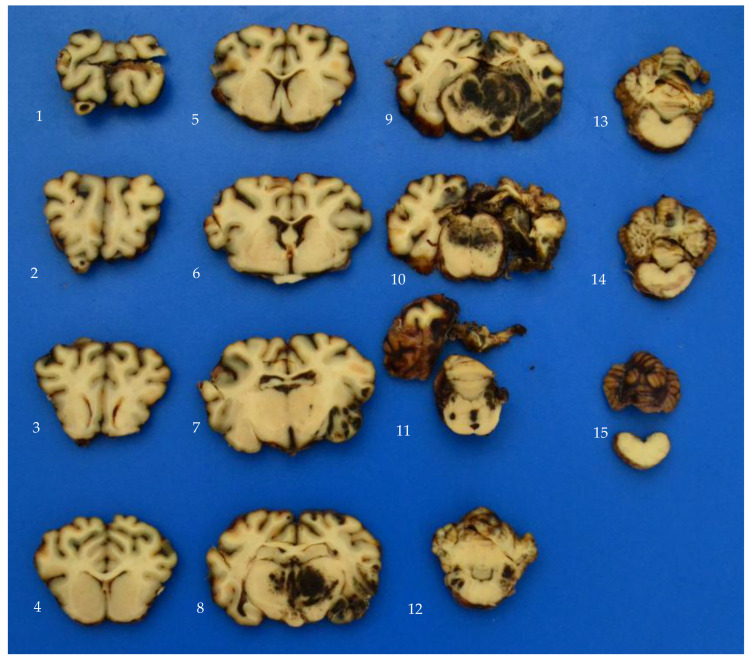
Fixed brain slices from a completely concussed polled ewe shot with a 0.22 Cash Special plus 2.5 gr cartridge. The slices are ordered sequentially from rostral (1) to caudal (15). Note the extensive haemorrhage throughout the thalamus and midbrain structures and in the left and right ventricles, 3rd ventricle and cerebral aqueduct. The damage to the frontal lobe in slice (1) is from extraction artefact.

Figure 3 shows an example of the fixed brain slices from a permanently concussed polled ewe, which was shot at the midline into the occipital lobe, with the bolt causing severe damage to the occipital lobes, right temporal lobe, thalamus, right optic tract, and midbrain reticular formation. There is extensive haemorrhage throughout the thalamus and midbrain structures and in the left and right ventricles, third ventricle and cerebral aqueduct. The maximum depth of injury was 36 mm.

**Figure 4:**
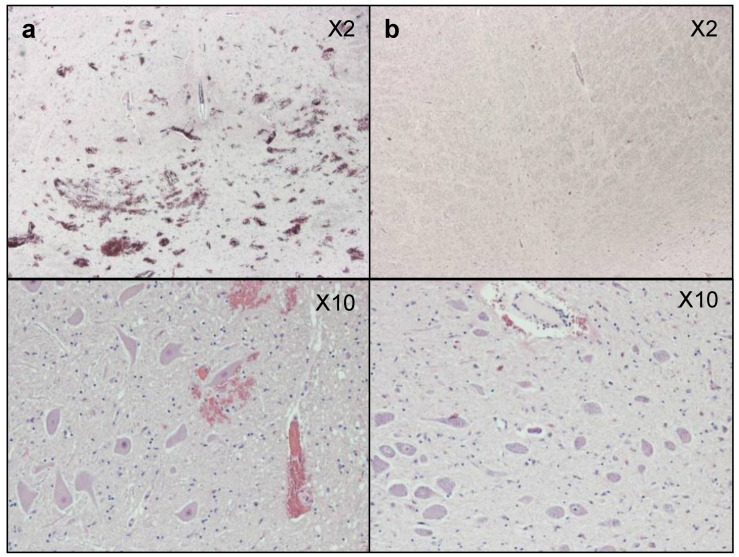
Brain slices from the level of the pons taken with either an ×2 or ×10 objective from (**a**) horned ewe with ruptured capillaries and (**b**) horned ewe with little haemorrhage.

Haemorrhage was more clearly identified from the gross fixed brain slices than from the MRI. Figure 4a,b show histological stained brain slices from the level of pons from two horned ewes at ×2 and ×10 magnification. The ewe in 4a shows obvious ruptured capillaries at both magnifications, while the animal in 4b has a limited number of ruptured capillaries. This type of haemorrhage could not be clearly identified in the MRI, even with prior gadolinium infusion. The sections of brain stem that were stained showed no signs of APP expression.

### 3.3. Factors Determining Incomplete Concussion

Polled ewes shot with any 1 gr configuration or with the point of entry >15 mm from bregma were more likely to present incomplete concussion; however, this was not significant (Table 5). A significant association was found between incomplete concussion and bolt penetration depth (*p* < 0.001), with ewes receiving a shot penetrating less than 37 mm being more likely to have incomplete concussion (Table 5).

**Figure 5:**
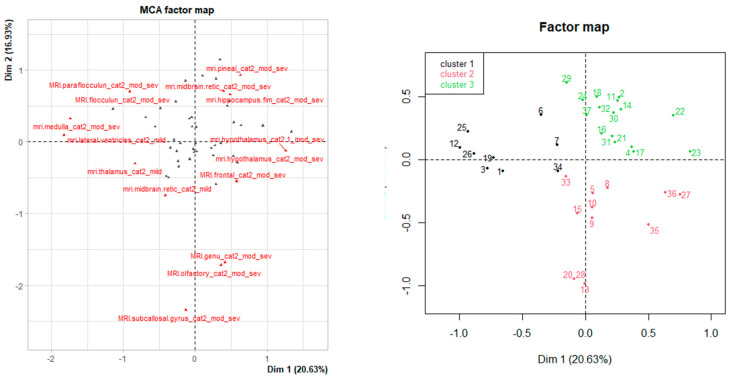
Results from MCA showing the distribution of the first 15 variables that are better represented in each dimension (**left**) and the distribution of clusters following HCA on the factor map (**right**).

Following MCA and HCA, ewes were classified into three clusters according to direct damage to specific brain structures following MRI examination, explaining 37.6% of the variance. Figure 5 and Supplementary Materials Tables S5 and S6 present the characteristics of each cluster. Variables that are further away from zero are better represented on the dimension than those close to zero, and variables close to each other on the factor map are closely related. Cluster 1 was characterised by moderate–severe damage in vermis, pons and medulla, and mild damage in various regions; cluster 2 was characterised by moderate–severe damage in frontal lobe, olfactory, corpus callosum genu, lateral ventricles, corpus callosum body, hypothalamus and subcallosal gyrus and mild damage in various regions; cluster 3 was characterised by moderate–severe damage in occipital and temporal lobes, third ventricle, thalamus, hippocampus fimbria, pineal, midbrain reticular formation, midbrain, ponds, corpus callosum splenium, hippocampus and cerebral aqueduct. Ewes belonging to clusters 1 and 2 were 3.5-times more likely to have incomplete concussion than those belonging to cluster 3 (Table 6); however, differences were not significant, which is likely to be due to the low number of animals presenting incomplete concussion and the relatively small sample size. Gross pathology classification was not distinctive enough, and no clear patterns or clusters were found following MCA and HCA.

## 4. Discussion

The results of this study demonstrate the significant role of kinetic energy transfer, bolt placement and penetration depth in inducing a state of irrecoverable unconsciousness in sheep shot with PCB. During PCB stunning, the kinetic energy of the fast-moving bolt is transferred to the sheep’s cranium, causing both focal and diffuse brain damage and disrupting cerebral function [3]. Focal damage in the current study was characterized by direct physical injury at the penetration site, in the wound path created by the bolt and paths of secondary missiles (bone, skin and wool fragments). Diffuse damage arising from shock waves, coup and contrecoup forces, shear forces and cerebral haemorrhaging was principally seen in the form of diffuse haemorrhage and ruptured capillaries distal to the wound path. The combination of extensive focal and diffuse trauma to cortical, thalamic and brainstem structures resulted in the loss of normal function and consciousness [3,15,16,20].

The results of this and previous studies indicate that the kinetic energy delivered to the cranium by the captive bolt is what induces immediate unconsciousness and insensibility [6,8]. Meanwhile, the irreversible unconsciousness is specifically due to the direct physical trauma to brain structures caused by the penetrating bolt [3]. In the present study, animals with less than 37 mm penetration were more likely to exhibit signs of incomplete concussion, further supporting that penetration depth is a crucial determinant of irrecoverable unconsciousness. Importantly, the variability in skull thickness and soft tissue depth between individual horned and polled ewes did not significantly affect the outcomes, indicating that bolt configuration and placement are more critical in ensuring effective stunning. This is further supported by the work of Gibson et al. [3], who showed no significant difference in PCB stunning performance between adult horned and polled ewes shot with the same PCB/cartridge combination. However, it was reported that the stunning of horned rams was more challenging than horned/polled ewes and polled rams [3], requiring increased powerloads to deliver the kinetic energy required to consistently render the horned rams irrecoverably unconscious.

The severity and location of brain damage varied in both horned and polled ewes, principally due to the different aiming positions and different PCB/cartridge configurations used. Of the animals that showed signs of incomplete concussion, two had macroscopic mild damage in the thalamus and pons region. Accounts from human TBI cases suggest that immediate unconsciousness can be produced with light manipulation or damage to specific brainstem structures, including the lamina terminalis, roof of the third ventricle, thalamus, pons, reticular formation and medulla [21]. However, conversely, in the current study, some animals with mild damage to thalamus, midbrain and pons continued to have rhythmic respiration and positive brainstem and cranial/spinal responses. This suggests that in sheep, some level of mild damage to these regions can be tolerated by the central nervous system (CNS) prior to unconsciousness and insensibility. In humans, it has been suggested that lesions confined to the ventral pons and dorsal medulla do not invariably cause alterations in consciousness [22,23].

Both PCB and NPCB cause a combination of macroscopic and microscopic trauma. Damage in the form of microscopic diffuse injury following PCB would be expected to be present and may depress consciousness. The expression of APP has been previously reported within the cerebral hemisphere, cerebellum and brainstem two hours after impact in PCB shot mechanically ventilated sheep [13]. In the current study, the stained brainstems showed no signs of APP expression. Amyloid precursor protein takes a minimum of 1 h for expression in brain tissue of ventilated PCB shot sheep [24]. It is likely that the period from injury to death and fixation was not sufficient to allow for the expression of APP.

An advantage of this study compared to those based in commercial abattoir conditions was the dual use of pathology assessment of PCB trauma with both MRI and perfused fixed macroscopic/microscopic techniques. This approach, when combined with assessment of the brainstem and cranial/spinal responses, provides a uniquely detailed picture of the locations of lesions/lacerations, the level of focal and diffuse tissue trauma disruption and the relating influence on behavioural and brainstem responses. This level of detail is often impossible to achieve in larger abattoir-based studies, where the conditions and numbers of animals assessed preclude the use of MRI and fixed pathology assessment due to cost and logistics. In these studies, often, the pathology is performed on frozen/case-hardened heads [3,12,15,16,25,26,27,28,29] or on fresh samples [30,31], where there is significant potential for distortion and displacement of tissues, as well as a lack of resolution, which can complicate assessment.

Magnetic resonance imaging provided a highly detailed snapshot of the in situ brain in the minutes immediately following penetrating captive bolt injury. This technique has only previously been used for the assessment of PCB stunning of water buffaloes/cattle [32] and goats [33] during PCB stunning; however, both those studies did not examine behavioural/brainstem/spinal responses. In the present study, MRIs were examined in the sagittal and transverse planes, allowing for the detailed assessment of injury without disruption of tissues and skin and bone fragments. It also provided a valuable opportunity to compare the findings from MRI scanning and those from gross pathology. In general, gross pathology tended to underestimate the frequency of moderate plus severe damage within the brain compared to MRI scanning. There was moderate to substantial agreement between MRI and gross fixed pathology for damage to specific brain structures, including the thalamus, midbrain, pons and medulla. However, for some structures, there was little to no agreement, an example being the midbrain reticular formation, with damage more identified with MRI than gross pathology. This structure is part of the wider reticular formation, which has an important role in the level of arousal and consciousness [20]. Lesions to this region or its projections to the cortex and thalamus (ascending reticular activating system) can result in unconsciousness in mammals [34].

There are several potential factors that may have influenced the level of agreement across different structures. Haemorrhage within the brain was more readily identified from the fixed gross pathology than from MRI, even with the pre-treatment application of the contrast agent (gadolinium). Possible explanations are that the resolution of the MRI was not sufficient to identify ruptured capillaries within specific brain structures; in some scans, there was ghosting of the image due to movement of the head, and during analysis of either the MRI or fixed brain slices, there could have been some false interpretation of haemorrhage as an artefact. Haemorrhage is more readily identified in situ from X-ray computed tomography (CT). CT has been previously used to characterise the level of PCB-induced haemorrhage and cranial fractures in piglets [35] and cranial factures alone in cattle and goats [32,33,36]. In the current experiment, the advantages of the MRI for soft tissue imaging were deemed more informative than the use of CT. In general, it was difficult to accurately match the fixed brain slice with the corresponding MRI transverse slices during analysis. The MRI slices were 3.5 mm thick with a 0.3 mm interspace. When slicing the fixed brains, the intention was to produce 4 mm slices; however, it was difficult to consistently achieve this level of accuracy and prepare slices which corresponded precisely with the MRI. The thickness of the slice was often determined by the presence or absence of bone fragments and the need to minimise their displacement.

The MRI compared to gross pathology more clearly demonstrated the phenomenon of brain tissue extrusion through the hole in the cranium created by the captive bolt. This extrusion appeared to be linked to the severe displacement of neural structures, depending on the shot position. This phenomenon has been previously described in horned and polled ewes and rams [3] and alpacas [16] following PCB. It is likely that this phenomenon is caused by the combination of the suction-like effect of the bolt’s withdrawal [3,37], increased intracranial pressure from haemorrhage/cerebrospinal fluid leakage from the subarachnoid space and ventricles [38], and the release of pressure caused by the withdrawal of the volume of the bolt [38]. In the current study, based on the MRI results, brain extrusion was present in almost all ewes, irrespective of whether they were completely or incompletely concussed. This suggests that extrusion of brain tissue from the bolt entrance hole immediately after the shot should not be used as a practical indicator of the level of brain trauma or shot effectiveness.

Although this study was conducted in controlled conditions, there were several factors that may have influenced the results. The sample size of the study was a limiting factor; this was unavoidable in a detailed study such as this due to logistic, ethical and financial pressures. The unbalanced sample sizes between treatments, which necessitated the grouping for statistical analysis, limited the ability to compare treatments. However, it should be noted this was unlikely to have influenced the central findings of the study, as it was not designed to compare treatments; instead, they were selected to produce a range of pathological states that would allow for the assessment of the pathophysiology of incomplete concussion. From a pathology perspective, although the brains were perfused and fixed in situ prior to removal from the cranial vault, the brains may have changed shape after removal because of gravity and the absence of support from the cranium and dura. Discrepancies may also arise from the process of brain removal and slicing, which could have dislodged bone, tissue (skin and wool) and haemorrhage fragments, complicating direct comparisons with MRI images. When fragments did become dislodged, particularly during slicing, it was often difficult to determine the original location and orientation. Furthermore, the fixed brain slices and MRI in some cases did not align perfectly due to differences in slice thickness and potential deformation of the brain post-fixation. These potential methodological-related variations and the observed differences between MRI and gross pathology emphasize the importance and advantage of a multi-diagnostic approach when assessing the extent of TBI following PCB stunning.

## 5. Conclusions

In conclusion, this study elucidates the critical factors influencing the efficacy of PCB-induced trauma in producing a state of irreversible unconsciousness in horned and polled sheep. It highlights the importance of delivered kinetic energy, bolt placement, penetration depth and severity of brain trauma to reduce the risks of incomplete concussion leading to animal welfare compromise. Furthermore, it shows that PCB can function as a single-step killing method for sheep, provided sufficient trauma is inflicted to the brain, particularly the thalamic and brainstem structures. This could be of practical importance if PCB was required to be used in the control of an infectious animal disease outbreak. Finally, this study shows that MRI gives a different perspective of PCB-induced TBI, providing detailed and accurate insights into the extent of damage, which aligns well with findings from gross pathology. These findings contribute valuable knowledge to the field of animal welfare, veterinary pathology and livestock management, enhancing the understanding of the mechanisms underlying effective PCB stunning.

## Figures and Tables

**Table 1:** Animal type, PCB/cartridge combination and numbers of animals shot with each combination.

Animal Type	Gun Type/Modification	Cartridge (Grain)	Number of Animals
Horned ewes	Cash Special	2.5 gr	11
	Cash Special	1 gr	1
	Cash Special spacer	1 gr	3
	Cash Special 1 mm travel and spacer	1 gr	3
Polled ewes	Cash special	2.5 gr	14
	Cash Special	1 gr	1
	Cash Special 1 mm travel and spacer	1 gr	4

**Table 2:** Skull and shot characteristics stratified by head type and PCB configuration.

		Head Type		
	Overall	Horned Ewes (*n* = 18)	Polled Ewes (*n* = 19)	*p* Value
**Skull characteristics** Soft tissue depth (mm)	6 (4–12)	6 (4–12)	6 (4–12)	0.62
Skull thickness (mm)	8 (6–25)	8 (7–12)	8 (6–10)	0.21
**Shot characteristics** Bolt penetration (mm)	36 (8–60)	35 (15–44)	36 (8–60)	0.54
Distance from Bregma 0 to 5 mm 6 to 15 mm 16 to 25 mm >25 mm	3 (8.1%) 14 (37.8%) 11 (29.7%) 9 (24.3%)	1 (6%) 10 (56%) 4 (22%) 3 (17%)	2 (11%) 4 (21%) 7 (37%) 6 (32%)	
		**PCB configurations**		
**Shot characteristics**	**Overall**	**1 gr any (*n* = 12)**	**2.5 gr (*n* = 25)**	***p* value**
Bolt penetration (mm)	36 (8–60)	21 (8–48)	40 (25–60)	**0.0003**

**Table 3:** Number (%) of brainstem and cranial/spinal responses for horned (*n* = 18) and polled (*n* = 19) (pooled) ewes shot with a 0.22 Cash Special with different cartridge combinations and PCB configurations.

	Spacer + 1 mm of Bolt Travel 1 gr Cartridge	Spacer 1 gr Cartridge	1 gr Cartridge	2.5 gr Cartridge
Number of animals	7	3	2	25
Failed to collapse	1 (14.3%)	0 (-)	0 (-)	0 (-)
Rhythmic breathing	4 (57.1%)	0 (-)	0 (-)	1 (4.0%)
Positive corneal reflex	3 (42.9%)	0 (-)	0 (-)	0 (-)
Positive palpebral reflex	3 (42.9%)	0 (-)	0 (-)	0 (-)
Eyeball rotated	3 (42.9%)	0 (-)	0 (-)	3 (12.0%)
Showing nystagmus	1 (14.3%)	0 (-)	0 (-)	1 (4.0%)
Not showing a relaxed jaw	2 (28.6%)	0 (-)	0 (-)	0 (-)

**Table 4:** Damage observed in brain regions and level of agreement between MRI and fixed gross pathology findings for distortion and direct physical damage using Kappa values.

Brain Region	Severity of Damage no. (%)						Kappa	
	Gross Pathology			MRI				
	Mild	Moderate	Severe	Mild	Moderate	Severe	Unweighted	Weighted
**Lobes of cerebrum**								
▪ Occipital	23 (62.2)	3 (8.1)	11 (29.7)	18 (48.6)	6 (16.2)	13 (35.1)	0.30	**0.47 ^‡^**
▪ Temporal	33 (89.1)	0 (-)	4 (10.8)	24 (64.9)	5 (13.5)	8 (21.6)	0.05	0.07
▪ Parietal	22 (59.5)	1 (2.7)	14 (37.8)	18 (48.6)	5 (13.5)	14 (37.8)	0.38	**0.49 ^‡^**
▪ Frontal	24 (64.9)	3 (8.1)	10 (27.0)	20 (54.0)	7 (18.9)	10 (27.0)	0.28	**0.47 ^‡^**
**Cerebellum**								
▪ Vermis	34 (91.9)	0 (-)	3 (8.1)	31 (83.8)	3 (8.1)	3 (8.1)	0.40	**0.54 ^‡^**
▪ Paraflocculun	33 (89.2)	0 (-)	4 (10.8)	29 (78.4)	4 (10.8)	4 (10.8)	0.44	**0.61 ^‡‡^**
▪ Folocculun	36 (97.3)	0 (-)	1 (2.7)	29 (78.4)	4 (10.8)	4 (10.8)	0.19	0.30
**Rostral to caudal**								
▪ Olfactory	36 (97.3)	0 (-)	1 (2.7)	33 (89.2)	2 (5.4)	2 (5.4)	0.38	**0.55 ^‡^**
▪ Corpus callosum genu	36 (97.3)	0 (-)	1 (2.7)	31 (83.8)	3 (8.1)	3 (8.1)	0.26	**0.39 ^†^**
▪ Lateral ventricles	18 (48.6)	1 (2.7)	18 (48.6)	6 (16.2)	6 (16.2)	25 (67.6)	0.26	**0.36 ^†^**
▪ Subcallosal gyrus	36 (97.3)	0 (-)	1 (2.7)	34 (91.9)	1 (2.7)	2 (5.4)	−0.04	−0.04
▪ Corpus callosum body	33 (89.2)	2 (5.4)	2 (5.4)	25 (67.6)	7 (18.9)	5 (13.5)	0.004	0.08
▪ 3rd Ventricle	32 (86.5)	4 (10.8)	1 (2.7)	27 (73.0)	3 (8.1)	7 (18.9)	0.09	0.02
▪ Corona radiata	35 (94.6)	1 (2.7)	1 (2.7)	35 (94.6)	1 (2.7)	1 (2.7)	0.22	**0.37 ^†^**
▪ Internal capsule	27 (72.9)	1 (2.7)	9 (24.3)	17 (45.9)	3 (8.1)	17 (45.9)	0.26	**0.31 ^†^**
▪ Thalamus	26 (70.3)	5 (13.5)	6 (16.2)	16 (43.2)	8 (21.6)	13 (35.1)	0.34	**0.32 ^†^**
▪ Hypothalamus	35 (94.6)	0 (-)	2 (5.4)	30 (81.1)	1 (2.7)	6 (16.2)	0.16	0.17
▪ Mammillary body	36 (97.3)	0 (-)	1 (2.7)	32 (86.5)	0 (-)	5 (13.5)	−0.05	−0.05
▪ Hippocampus fimbria	27 (73.0)	1 (2.7)	9 (24.3)	21 (56.8)	6 (16.2)	10 (27.0)	0.42	**0.59 ^‡^**
▪ Midbrain reticular formation	35 (94.6)	1 (2.7)	1 (2.7)	18 (48.6)	5 (13.5)	14 (37.8)	0.13	0.08
▪ Pineal	31 (83.8)	1 (2.7)	5 (13.5)	27 (73.0)	7 (18.9)	3 (8.1)	0.27	**0.32 ^†^**
▪ Corpus callosum splenium	33 (89.2)	2 (5.4)	2 (5.4)	29 (78.4)	3 (8.1)	5 (13.5)	0.25	**0.41 ^‡^**
▪ Hippocampus head	35 (94.6)	0 (-)	2 (5.4)	20 (54.1)	5 (13.5)	12 (32.4)	0.14	0.18
▪ Hippocampus fim	27 (73.0)	1 (2.7)	9 (24.3)	21 (56.8)	6 (16.2)	10 (27.0)	0.42	**0.59 ^‡^**
▪ Midbrain	28 (75.7)	5 (13.5)	4 (10.8)	29 (78.4)	3 (8.1)	5 (13.5)	0.36	**0.48 ^‡^**
▪ Cerebral aqueduct	35 (94.6)	2 (5.4)	0 (-)	32 (86.5)	2 (5.4)	3 (8.1)	0.07	0.09
▪ Pons	36 (97.3)	1 (2.7)	0 (-)	32 (86.5)	2 (5.4)	3 (8.1)	0.14	**0.24 ^†^**
▪ Medulla	35 (94.6)	1 (2.7)	1 (2.7)	32 (86.5)	1 (2.7)	4 (10.8)	0.39	**0.51 ^‡^**

^†^ 0.20–0.40 fair agreement; ^‡^ 0.41–0.60—moderate agreement; and ^‡‡^ 0.61–0.80—substantial agreement.

**Table 5:** Distribution of ewe and shot factors considered for incomplete concussion following reclassification and univariable analysis (*n* = 37).

	Incomplete Concussion		
	No	Yes	*p* Value
**Ewe heads characteristics**			
*Head type* Horned Polled	Number (%) 16 (88.9) 12 (63.2)	Number (%) 2 (11.1) 7 (36.8)	0.12
*Skull thickness* (mm)	Median (min–max) 8 (6–16)	Median (min–max) 8 (6–25)	0.36
*Soft tissue depth* (mm)	6 (4–12)	6 (5–12)	0.29
**Shot characteristics**			
*PCB configuration* 1 grain all configurations † 2.5 grain	Number (%) 6 (50%) 22 (88.0)	Number (%) 6 (50%) 3 (12.0)	0.14
*Point of entry (related to bregma)*			
0 to 5 mm 6 to 15 mm 16 to 25 mm >25 mm *Point of entry (related to bregma—2 categories)* Up to 15 mm >15 mm	3 (100.0) 12 (85.7) 8 (72.7) 5 (55.6) 15 (88.2) 13 (65.0)	0 (-) 2 (14.3) 3 (27.3) 4 (44.4) 2 (11.8) 7 (35.0)	0.38 0.14
Midline shift * Absent Present	11 (73.3) 15 (78.9)	4 (26.7) 4 (21.1)	1
*Bolt penetration (mm)*	Median (min-max) 40 (21–60)	Median (min-max) 18 (8–40)	**<0.001**
*Bolt penetration depth (2 categories)* 8 to 36 mm 37 to 60 mm	12 (60.0) 16 (94.1)	8 (40.0) 1 (5.9)	**0.02**

* Not recorded in three animals. † Includes inclusion of muzzle spacer and 1 mm of bolt travel.

**Table 6:** Results of logistic regression model assessing the likelihood of incomplete concussion in each of the clusters identified by hierarchical cluster analysis, according to direct damage to specific brain structures following MRI examination.

Cluster	Incomplete Concussion		OR (95% C.I)	*p* Value
	No Number (%)	Yes Number (%)		
C3 (*n* = 16) C1 (*n* = 9)	14 (87.5) 6 (66.7)	2 (12.5) 3 (33.3)	*Ref* 3.5 (0.47–32.3)	0.198
C2 (*n* = 12)	8 (66.7)	4 (33.3)	3.5 (0.55–29.6)	0.226

## Data Availability

All data are contained within this manuscript and Supplementary Materials.

## Supplementary Materials

The following supporting information can be downloaded at: https://www.mdpi.com/article/10.3390/vetsci12010053/s1, Table S1: Number of brainstem and cranial/spinal responses for polled ewes (*n* = 19) shot with the 0.22 Cash Special with different cartridge combinations and PCB configurations; Table S2: Number of brainstem and cranial/spinal responses for horned ewes (*n* = 19) shot with the 0.22 Cash Special with different cartridge combinations and PCB configurations; Table S3: Signs of recovery in individual horned ewes shot with a 0.22 Cash Special in different configurations and severity of brain damage to specific global structures, determined from MRI and fixed gross pathology; + Mild, ++ Moderate, +++ Severe. Animals in bold and shaded were classed as conscious/sensible; Table S4: Signs of recovery in individual polled ewes shot with a 0.22 Cash Special in different configurations and severity of brain damage to specific global structures, determined from MRI and fixed gross pathology; + Mild, ++ Moderate, +++ Severe. Animals in bold and shaded were classed as conscious/sensible; Table S5: Features of the three clusters identified after MCA and HCA using MRI damage classification for lobes of the cerebrum and cerebellum; Table S6: Features of the three clusters identified after MCA and HCA using MRI damage classification for brain regions rostral to caudal, exclude the cerebrums and cerebellum.
