# Supplementary material for: Pathophysiology of Penetrating Captive Bolt Stunning in Horned and Polled Sheep and Factors Determining Incomplete Concussion
Source: Vet Sci. 2025 Jan 13;12(1):53. doi: 10.3390/vetsci12010053 (PMC11769382; doi:10.3390/vetsci12010053)
Supplement: Supplementary file 1 [file vetsci-12-00053-s001.zip › vetsci-3388177-supplementary.pdf]

Supplementary material

Table S1. Number of brainstem and cranial/spinal responses for polled ewes (n=19) shot with the .22 Cash Special with different cartridge combinations and PCB configurations.

	**Spacer + 1mm of bolt travel 1gr cartridge**	**1gr cartridge**	**2.5gr purple cartridge**
Number of animals	4	1	14
Failed to Collapse	1	0	0
Rhythmic breathing	3	0	1
Positive corneal reflex	2	0	0
Positive palpebral reflex	2	0	0
Eyeball rotated	2	0	3
Showing nystagmus	1	0	1
Not showing a relaxed jaw	1	0	0

Table S2. Number of brainstem and cranial/spinal responses for horned ewes (n=19) shot with the .22 Cash Special with different cartridge combinations and PCB configurations.

	**Spacer + 1mm of bolt travel 1gr cartridge**	**Spacer 1gr cartridge**	**1gr cartridge**	**2.5gr purple cartridge**
Number of animals	3	3	1	11
Failed to collapse	0	0	0	0
Rhythmic breathing	1	0	0	0
Positive corneal reflex	1	0	0	0
Positive palpebral reflex	1	0	0	0
Eyeball rotated	1	0	0	0
Showing nystagmus	0	0	0	0
Not showing a relaxed jaw	1	0	0	0

Table S3. Signs of recovery in individual horned ewes shot with a .22 Cash Special in different configurations and severity of brain damage to specific global structures, determined from MRI and fixed gross pathology.

Animal	Signs of recovery	Cartridge	CBG configuration	Lobes of Cerebrums					Midbrain	Pons	Medulla	Cerebellum
				Occipital	Temporal	Parietal	Frontal	Thalamus				
1	No	2.5gr	standard			++	+	++				
2	No	2.5gr	standard		++	++	+	++	+			
3	No	2.5gr	standard		+	+++	+	+++				
4	No	2.5gr	standard					+++	+++			++
5	No	2.5gr	standard	+++						+	+++	+++
6	No	2.5gr	standard							+++	+++	+++
7	No	2.5gr	standard	+++	+				++	+++	+	
8	No	2.5gr	standard					+++	++			++
9	No	2.5gr	standard	+++		++		+++	++	+		
10	No	2.5gr	standard			+	+++					
11	No	2.5gr	standard			+	+++	++				
12	No	1gr	standard	+		+		+++	+++	+		
13	No	1gr	spacer					+++				++
14	No	1gr	spacer	+		+++			+			+
15	No	1gr	spacer			+++	+	+++	+			+
**16**	**Yes**	**1gr**	**travel and spacer**			**++**	**++**					
17	No*	1gr	travel and spacer	+++	+				+	+		+
18	No	1gr	travel and spacer	+++	+			+	+	+		++

* Animal displayed a shallow depth of concussion with eyeball rotation but did not recover.

+ Mild, ++ Moderate, +++ Severe.

Animals in bold and shaded were classed as conscious/sensible.

Table S4. Signs of recovery in individual polled ewes shot with a .22 Cash Special in different configurations and severity of brain damage to specific global structures, determined from MRI and fixed gross pathology.

Animal	Signs of recovery	Cartridge	CBG configuration	Lobes of Cerebrums				Thalamus	Midbrain	Pons	Medulla	Cerebellum
				Occipital	Temporal	Parietal	Frontal					
1	No	1gr	standard			+++	+	++	+++	+++		++
**2**	**Yes**	**1gr**	**travel and spacer**			**+++**	**+++**	**+**				
**3**	**Yes**	**1gr**	**travel and spacer**	**+**								**+**
**4**	**Yes**	**1gr**	**travel and spacer**		**+++**	**+++**	**+**					
**5**	**Yes**	**1gr**	**travel and spacer**				**+**					
6	No	2.5gr	standard	+++	+++			+++	++			
7	No	2.5gr	standard		++	+	+++	+++	++	+	+	++
**8**	**No†**	**2.5gr**	**standard**	**+++**	**+++**			**++**	**++**			
9	No	2.5gr	standard	+++	+++	+++	+++	+++	++			
10	No	2.5gr	standard	+++	+++		+	+	+			++
11	No	2.5gr	standard	+++		+++			+			+
**12**	**Yes**	**2.5gr**	**standard**	**+++**	**+++**				**+**	**+**		**++**
13	No	2.5gr	standard			+++		+++	+			+
14	No	2.5gr	standard			+	+++	+++	+			
15	No*	2.5gr	standard	+						+	+	+++
16	No	2.5gr	standard	+++						+	+++	+++
17	No	2.5gr	standard	+++					+	+	+++	+++
18	No	2.5gr	standard				+++					
19	No	2.5gr	standard	+	+++		+++	+++	+	+		+

* Animal displayed a shallow depth of concussion with eyeball rotation but did not recover.

† Animal had eyeball rotation and nystagmus.

+ Mild, ++ Moderate, +++ Severe.

Animals in bold and shaded were classed as conscious/sensible.

Table S5. Features of the three clusters identified after MCA and HCA using MRI damage classification for lobes of the cerebrum and cerebellum.

Brain region	Clusters identified		
	C1 (n=9)	C2 (n=12)	C3 (n=16)
**Lobes of cerebrum**			
Occipital			
Mild	1 (5.5)	12 (66.6)	5 (27.8)
Moderate & Severe	8 (42.1)	0 (-)	11 (57.9)
Temporal			
Mild	7 (29.2)	9 (37.5)	8 (33.3)
Moderate & Severe	2 (15.4)	3 (23.1)	8 (61.5)
Parietal			
Mild	8 (44.4)	3 (16.7)	7 (38.9)
Moderate & Severe	1 (5.3)	9 (47.4)	9 (47.4)
Frontal			
Mild	9 (45.0)	1 (5.0)	10 (50.0)
Moderate & Severe	0 (-)	11 (64.7)	6 (35.3)
**Cerebellum**			
Vermis			
Mild	5 (16.1)	12 (38.7)	14 (45.2)
Moderate & Severe	4 (66.7)	0 (-)	2 (33.3)
Paraflocculus			
Mild	5 (17.2)	12 (41.4)	12 (41.4)
Moderate & Severe	4 (50.0)	0 (-)	4 (50.0)
Folocculun			
Mild	5 (17.2)	12 (41.4)	12 (41.4)
Moderate & Severe	4 (50.0)	0 (-)	4 (50.0)

Table S6. Features of the three clusters identified after MCA and HCA using MRI damage classification for brain regions rostral to caudal, exclude the cerebrums and cerebellum.

Brain regions rostral to caudal	Clusters identified		
	C1 (n=9)	C2 (n=12)	C3 (n=16)
Olfactory bulbs			
Mild	9 (27.3)	9 (27.3)	15 (93.7)
Moderate & Severe	0 (-)	3 (75.0)	1 (25.0)
Corpus callosum genu			
Mild	9 (29.0)	6 (19.4)	16 (51.6)
Moderate & Severe	0 (-)	6 (100)	0 (-)
Lateral ventricles			
Mild	6 (100)	0 (-)	0 (-)
Moderate & Severe	3 (9.7)	12 (38.7)	16 (51.6)
Subcallosal gyrus			
Mild	9 (27.5)	9 (26.5)	16 (47.0)
Moderate & Severe	0 (-)	3 (100)	0 (-)
Corpus callosum body			
Mild	9 (36.0)	7 (28.0)	9 (36.0)
Moderate & Severe	0 (-)	5 (41.7)	7 (58.3)
3rd Ventricle			
Mild	9 (33.3)	9 (33.3)	9 (33.3)
Moderate & Severe	0 (-)	3 (30.0)	7 (70.0)
Corona radiata			
Mild	9 (25.7)	11 (31.4)	15 (42.9)
Moderate & Severe	0 (-)	1 (50.0)	1 (50.0)
Internal capsule			
Mild	9 (52.9)	2 (11.8)	6 (35.3)
Moderate & Severe	0 (-)	10 (50.0)	10 (50.0)
Thalamus			
Mild	9 (56.2)	5 (31.3)	2 (12.5)
Moderate & Severe	0 (-)	7 (33.3)	14 (66.7)
Hypothalamus			
Mild	9 (30.0)	9 (30.0)	12 (40.0)
Moderate & Severe	0 (-)	3 (42.9)	4 (57.1)
Mammillary body			
Mild	9 (28.1)	10 (31.3)	13 (40.6)
Moderate & Severe	0 (-)	2 (40.0)	3 (60.0)
Hippocampus fimbria			
Mild	7 (33.3)	11 (52.4)	3 (14.3)
Moderate & Severe	2 (12.5)	1 (6.3)	13 (81.3)
Midbrain reticular formation			
Mild	7 (38.9)	11 (61.1)	0 (-)
Moderate & Severe	2 (10.5)	1 (5.3)	16 (84.2)
Pineal			
Mild	9 (33.3)	12 (44.4)	6 (22.2)
Moderate & Severe	0 (-)	0 (-)	10 (100)
Corpus callosum splenium			
Mild	8 (27.6)	12 (41.4)	9 (31.0)
Moderate & Severe	1 (12.5)	0 (-)	7 (87.5)
Hippocampus head			
Mild	8 (40.0)	10 (50.0)	2 (10.0)
Moderate & Severe	1 (5.9)	2 (11.8)	14 (82.4)
Hippocampus fim			
Mild	7 (33.3)	11 (52.4)	3 (14.3)
Moderate & Severe	2 (12.5)	1 (6.3)	13 (81.3)
Midbrain			
Mild	8 (27.6)	12 (41.4)	9 (31.0)
Moderate & Severe	1 (12.5)	0 (-)	7 (87.5)
Cerebral aqueduct			
Mild	8 (25.0)	12 (37.5)	12 (37.5)
Moderate & Severe	1 (20.0)	0 (-)	4 (80.0)
Pons			
Mild	7 (21.9)	12 (37.5)	13 (40.6)
Moderate & Severe	2 (40.0)	0 (-)	3 (60.0)
Medulla			
Mild	4 (12.5)	12 (37.5)	16 (50.0)
Moderate & Severe	5 (100)	0 (-)	0 (-)
